# Bcs1, a novel target for fungicide

**DOI:** 10.3389/fchem.2023.1146753

**Published:** 2023-03-13

**Authors:** Jingyu Zhan, Di Xia

**Affiliations:** Laboratory of Cell Biology, Center for Cancer Research, National Cancer Institute, National Institutes of Health, Bethesda, MD, United States

**Keywords:** Bcs1, Complex III, cytochrome bc_1_ complex, iron-sulfur protein, respiratory chain, AAA protein

## Abstract

The mitochondrial respiratory chain has long been a primary target for the development of fungicides for its indispensable role in various cellular functions including energy metabolism. Over the years, a wide range of natural and synthetic fungicides and pesticides targeting the respiratory chain complexes have been discovered or developed and used in agriculture and in medicine, which brought considerable economic gains but was also accompanied by the emergence of resistance to these compounds. To delay and overcome the onset of resistance, novel targets for fungicides development are actively being pursued. Mitochondrial AAA protein Bcs1 is necessary for the biogenesis of respiratory chain Complex III, also known as cyt *bc*_1_ complex, by delivering the last essential iron-sulfur protein subunit in its folded form to the cyt *bc*_1_ precomplex. Although no report on the phenotypes of knock-out Bcs1 has been reported in animals, pathogenic Bcs1 mutations cause Complex III deficiency and respiratory growth defects, which makes it a promising new target for the development of fungicides. Recent Cryo-EM and X-ray structures of mouse and yeast Bcs1 revealed the basic oligomeric states of Bcs1, shed light on the translocation mechanism of its substrate ISP, and provided the basis for structure-based drug design. This review summarizes the recent progress made on understanding the structure and function of Bcs1, proposes the use of Bcs1 as an antifungal target, and provides novel prospects for fungicides design by targeting Bcs1.

## 1 Introduction

### 1.1 Mitochondrial respiratory chain components as effective drug targets

Mitochondrial respiratory chain components are validated targets for disease treatment in medicine, and fungal and pest controls in agriculture by natural or synthetic compounds (Fisher et al., 2020). The respiratory Complex III, for example, has been targeted for treatment of malarial parasites and Pneumocystis jiroveci [Pneumocystis carinii] pneumonia with atovaquone (Baggish and Hill, 2002). Complex III is also widely targeted by antifungals in agriculture with synthetic azoxystrobin and famoxadone (Gao et al., 2002; Esser et al., 2004; Esser et al., 2019). The effective disease control by targeting the respiratory chain components reflects their fundamental and indispensable role in cellular function. However, like all chemical therapies, resistance to these treatments is inevitable, rendering drugs useless. Studies of resistance mechanisms indicated that pathogenic organisms are equipped with a plethora of tools to evade attacks by drugs. These tools include mutations to target drug binding site, deploying a large number of ABC transporters that have broad substrate specificity on the cell surface to fend off invading drugs, reducing the intracellular concentration of the drug by degradation, chemical modification, and storage into intracellular vesicles, and going into hibernation (Prasad et al., 2016). To circumvent or delay the onset of resistance, various approaches have been used such as designing derivatives of existing compounds, using a combination of different drugs against several targets simultaneously, and identifying new targets.

### 1.2 New targets are needed to overcome drug resistance

Despite the effort, drug resistance remains a major issue in treating microbial infections. New targets are actively being sought after. Identification of new targets is by no means easy because the targets must be verified extensively for its lethality to the pathogens and its safety to the hosts. One approach to efficiently identify new targets is to find proteins that are connected and essential to the validated targets, which includes protein assembly factors, translocations factors, and quality control factors. Inactivation of these factors adversely affect the structural and functional integrity of validated targets, thus achieving the goal of disease control. In this article, we review a mitochondrial protein factor Bcs1 that is essential to the assembly of respiratory Complex III and its potential as a drug target.

### 1.3 Cellular protein translocation pathways that are required for Complex III assembly

Nearly all human proteins are encoded by the nuclear genes and synthesized in the cytosol, except for 13 proteins that are encoded by mitochondrial genome and produced by the mitochondrial protein synthesis machinery (Boengler et al., 2011). A significant portion of nuclear-encoded proteins has to be either translocated across or inserted into the membrane through complex and energy-dependent translocation machineries. Most proteins are exported in unfolded states through the universal Sec pathway to their designated locations where they subsequently become folded to acquire their functional states (Park and Rapoport, 2012). As the main route for protein translocation, components of the Sec apparatus are evolutionarily conserved, as found in Sec61 in eukaryotes (Deshaies and Schekman, 1987) and SecYEG complex in bacteria (Bieker et al., 1990) and chloroplasts (Laidler et al., 1995). Nuclear encoded mitochondrial proteins are imported as unfolded precursors *via* the TOM (translocase of the outer membrane) and TIM (translocase of the inner membrane) complexes (Wasilewski et al., 2017). However, some proteins need to be translocated in their folded states, which in turn requires specialized translocons. This can happen when specific cofactors (Brandt et al., 1993; Lill, 2009) or metal ions (Tottey et al., 2008) have to be inserted into the protein active sites before translocation, or when a multi-subunit protein complex is directed to the translocation system by the signal sequence only present in one subunit of the complex (Sauve et al., 2007). In bacteria, this highly unusual task of transporting folded protein is carried out by the twin arginine translocation (Tat) pathway (Palmer and Berks, 2012). For example, some bacterial periplasmic proteins contain non-covalently bound redox cofactors that can only be inserted in the cytosol prior to translocation, and they are exported to the periplasm by the Tat system in the folded form with the cofactor installed.

Unlike Sec pathway, the Tat system is not ubiquitous, found in most, but not all bacteria and archaea (Palmer and Berks, 2012). The Tat system is also found in the thylakoid membrane of chloroplasts in plants and is essential for membrane translocation of many substrates including the assembly of the cytochrome cyt *b*_6_ *f* complex in the photosynthetic pathway by translocating the folded Rieske iron-sulfur protein (ISP) with the iron-sulfur cluster (Fe_2_S_2_) installed (Kurisu et al., 2003). The Tat apparatus is broadly conserved between prokaryotes and chloroplasts, and requires minimally three integral membrane proteins to function: TatA, TatB and TatC (Rollauer et al., 2012; Ramasamy et al., 2013; Rodriguez et al., 2013; Zhang et al., 2014; Palmer and Stansfeld, 2020). The Tat pathway substrates share a consensus signal sequence at the N-terminus featuring a critical pair of consecutive Arginine residues, which is recognized by TatC, the core component the Tat system (Ramasamy et al., 2013). Substrate translocation by the Tat apparatus is fueled by the cross-membrane proton motive force (PMF). Although the structure for the TatC pore (Rollauer et al., 2012; Ramasamy et al., 2013) and the helical regions of TatA (Rodriguez et al., 2013) and TatB (Zhang et al., 2014) have been experimentally determined individually, the stoichiometry of these components in the active Tat complex is not established and the structure of the substrate-activated Tat complex is not known. The mechanism by which folded substrates with various sizes get translocated across biomembranes without compromising membrane integrity remains elusive. By contrast, the Tat pathway is no longer preserved in fungal and animal mitochondria, although the need to translocate the folded ISP subunit for the assembly of the respiratory chain cyt *bc*_1_ complex (also known as Complex III), a homolog to the cyt *b*_6_ *f* in mitochondria, is still present. It became clear a decade ago that an AAA (ATPases Associated with diverse cellular Activities) protein Bcs1 was identified to perform this role in mitochondria in substitution for the Tat pathway (Fernandez-Vizarra et al., 2007; Wagener et al., 2011).

## 2 Function of mitochondrial AAA protein Bcs1

### 2.1 Bcs1 translocates folded ISP across the mitochondrial inner membrane for the assembly of cyt bc_1_ complex

**FIGURE 1:**
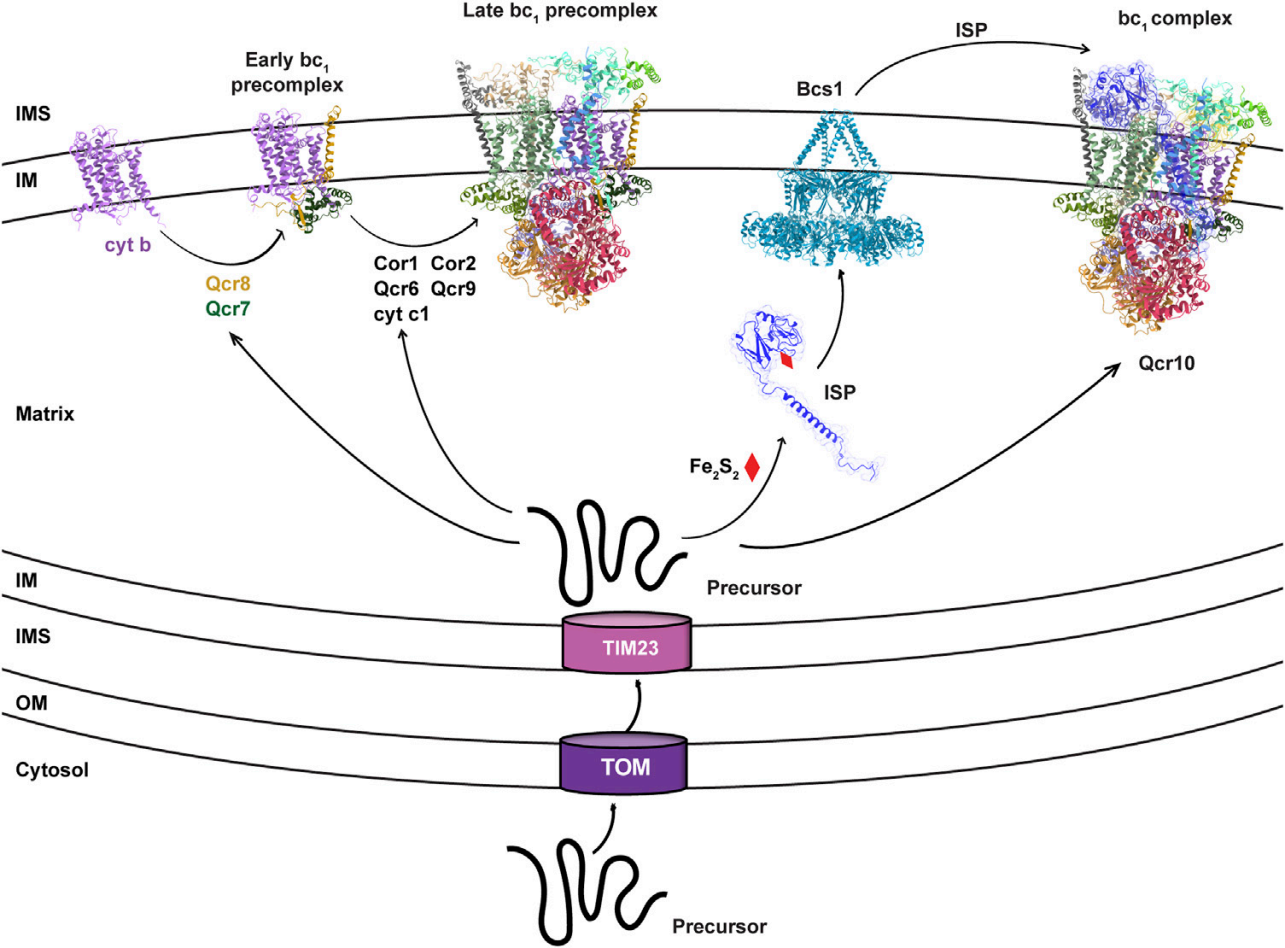
Bcs1 is involved in the late stage of bc_1_ complex biogenesis. The biogenesis of yeast bc_1_ complex (3CX5.pdb) is shown here to represent the overall process for eukaryotic bc_1_ complex. It starts with the translation of mitochondrial DNA encoded cyt b. *Via* stepwise addition of several non-catalytic subunits and catalytic cyt c1, the biogenesis of bc_1_ proceeds from early precomplex to late precomplex. ISP is the last catalytic subunit to be assembled into the bc_1_ complex, which is delivered in its folded form to bc_1_ precomplex by the translocase Bcs1.

The cyt *bc*_1_ complex is the central segment of the respiratory chain that performs reaction of electron transfer from NADH to molecular oxygen, and couples this reaction to proton pumping across the mitochondrial inner membrane (IM), creating a Proton Motive Force (PMF) across the membrane that fuels ATP synthesis and is used by many other cellular activities. Depending on the biological complexity, cyt bc_1_ complex can have as few as the only three essential subunits in bacteria (Yang and Trumpower, 1986), or up to 11 subunits in vertebrates (Xia et al., 1997). The three essential subunits are cytochrome *c*_1_ (cyt *c*_1_, or Cyt1 in yeast), cytochrome *b* (cyt *b*), and the Rieske iron-sulfur protein (ISP or Rip1 in yeast), all equipped with redox cofactors and involved in electron transfer. For eukaryotic cyt *bc*_1_ complex, cyt *b* is the only subunit that is coded by the mitochondrial DNA and plays a nucleating role for the cyt *bc*_1_ complex biogenesis (OBrien et al., 2009; Needs et al., 2021). Upon translation in mitochondrial matrix, the nascent cyt *b* peptide is co-translationally inserted into the IM, and the redox cofactor is subsequently installed from the matrix side (Smith et al., 2012). Nuclear-encoded cyt *c*_1_ is imported into the matrix *via* the TOM-TIM23 complex as an unfolded precursor (Neupert, 2015), which matures through IM insertion and heme *c*_1_ co-factor incorporation at intermembrane space (IMS) (Smith et al., 2012). Cyt *b*, cyt *c*_1_ and other non-catalytic subunits, also called supernumerary subunits, form the core of the bc_1_ precomplex, which is still inactive due to the lack of the essential subunit ISP (Figure 1).

ISP is the third catalytic subunit of cyt bc_1_ complex, with a N-terminal unstructured random coil integrated into the core region of the cyt *bc*_1_ complex, a middle transmembrane helix segment anchored to the IM, and a globular C-terminal domain (ISP-ED, extrinsic domain) remaining in the IMS (Xia et al., 1997). The iron-sulfur cluster (Fe_2_S_2_) is embedded in the ISP-ED and mediates electron transfer between ubiquinol and cyt *c*_1_ (Xia et al., 1997). Due to the matrix localization of the Fe_2_S_2_ cluster biosynthesis machinery (Lill, 2009), nuclear encoded ISP is first imported into the mitochondrial matrix as a precursor peptide *via* the TOM-TIM23 complex (Neupert, 2015), which subsequently becomes folded upon installation of the Fe_2_S_2_ cluster in the matrix. Then, the fully folded ISP-ED of the ISP precursor, stabilized additionally by a matrix protein Mzm1 (Cui et al., 2012), is translocated across the IM to IMS for the assembly of the cyt *bc*_1_ complex (Figure 1). In bacteria and plant chloroplasts where the Tat system is preserved, folded ISP-ED is exported to the periplasm and the intrathylakoid space, respectively, by the Tat system (Aldridge et al., 2008). In mitochondria, the AAA protein Bcs1 is shown to replace the Tat system to perform this role.

As the central component of the respiratory chain, the cyt *bc*_1_ complex has long been a popular target for the development of antibiotics (Fisher et al., 2020). Natural compounds such as strobilurin (Gewehr and Sauter, 2019) and synthetic compounds such as azoxystrobin (Esser et al., 2004; Esser et al., 2019) have been used world-wide as agricultural fungicides, which brought enormous economic gains. However, there are major concerns about these fungicides over emerging drug resistance (Lucas et al., 2015), as well as the toxicity towards the non-target terrestrial animals and aquatic lives. The sequence and structure of the catalytic subunits in cyt *bc*_1_ complex are highly conserved throughout evolution (Table 1), which makes it very difficult to design inhibitors with high selectivity. Furthermore, increasing number of fungal pathogens are developing resistance to the current inhibitors by evolving mutations in the drug target, which gets selected and amplified upon fungicide usage (Lucas et al., 2015). To delay and overcome the onset of resistance, novel targets with potentially better inhibitor specificity for fungicides development are needed.

**TABLE 1:** Sequence conservation to human homologue.

		Human	Bovine	Mouse	Zebrafish	Yeast	Malaria parasite
Bcs1	Uniprot	Q9Y276	Q5E9H5	Q9CZP5	Q7ZV60	P32839	A0A564ZXW0
	Identity	100	94.03	94.02	76.85	50.00	38.46
ISP	Uniprot	P47985	P13272	Q9CR68	Q6PC48	P08067	Q8IL75
	Identity	100	90.15	88.69	69.34	55.38	40.93
cyt c1	Uniprot	P08574	P00125	Q9D0M3	Q3B7R0	P07143	Q8I6U9
	Identity	100	92.62	90.46	69.69	52.98	51.29
cyt b	Uniprot	P00156	P00157	P00158	Q9MIX8	P00163	G1CTE5
	Identity	100	78.89	78.57	70.26	50.27	42.35

### 2.2 Bcs1 mutations are linked to cyt bc_1_ complex deficiencies

Bcs1 was first identified as an integral membrane protein in yeast mitochondria, for its indispensable role in incorporating ISP subunit into cyt *bc*_1_ complex (Nobrega et al., 1992). Bcs1 mutant in yeast led to a selective loss of ISP subunit in cyt *bc*_1_ complex and consequent enzymatic defect, which could be rescued/reverted by overexpressing Bcs1 but not ISP (Nobrega et al., 1992; Wagener et al., 2011). Further investigations into the ISP biogenesis pathway found that Bcs1 acts as a translocase that could recognize and translocate folded ISP-ED across the mitochondrial IM for it to assemble into the cyt *bc*_1_ complex (Wagener et al., 2011). Orthologs of Bcs1 were identified in nearly all eukaryotes, and similar cyt *bc*_1_ complex deficiencies were found to be linked with Bcs1 ortholog mutants (De Meirleir et al., 2003).

Human ortholog to Bcs1 is encoded by BCS1L (Bcs1-like) gene, and pathogenic BCS1L variants cause autosomal recessive inheritance with different prognoses (Hinson et al., 2007; Olahova et al., 2019). Infants born with the GRACILE syndrome show characteristic symptoms including growth retardation, aminoaciduria, cholestasis, iron overload, lactic acidosis, and early death. Because of its severeness, newborns develop liver and kidney failure quickly and could not survive for over a few months (Akduman et al., 2016; Guo et al., 2022). In milder cases such as the Björnstad syndrome, reported phenotypes include twisted hair shafts (pili torti) and hearing loss (Hinson et al., 2007). Clinical manifestations caused by pathogenic BCS1L variants can land anywhere between the two extremes.

Among various mitochondria diseases, BCS1L mutations remain to be the most frequent causes of mitochondrial respiratory chain deficiency (Fernandez-Vizarra and Zeviani, 2015), which in turn validates the indispensable role Bcs1 plays in the assembly of the cyt *bc*_1_ complex. If the Bcs1 activity of a eukaryotic pathogen could be selectively inhibited, the growth of the pathogen would be severely impaired due to the loss of cyt *bc*_1_ function, making Bcs1 a potential target for eukaryotic pathogens including fungus. The recent structural studies of mouse and yeast Bcs1 (Kater et al., 2020; Tang et al., 2020; Xia, 2021) revealed differences that laid the foundation to identify vulnerabilities in the enzyme suitable for structure-based design of selective drugs.

## 3 Structure of Bcs1

**FIGURE 2:**
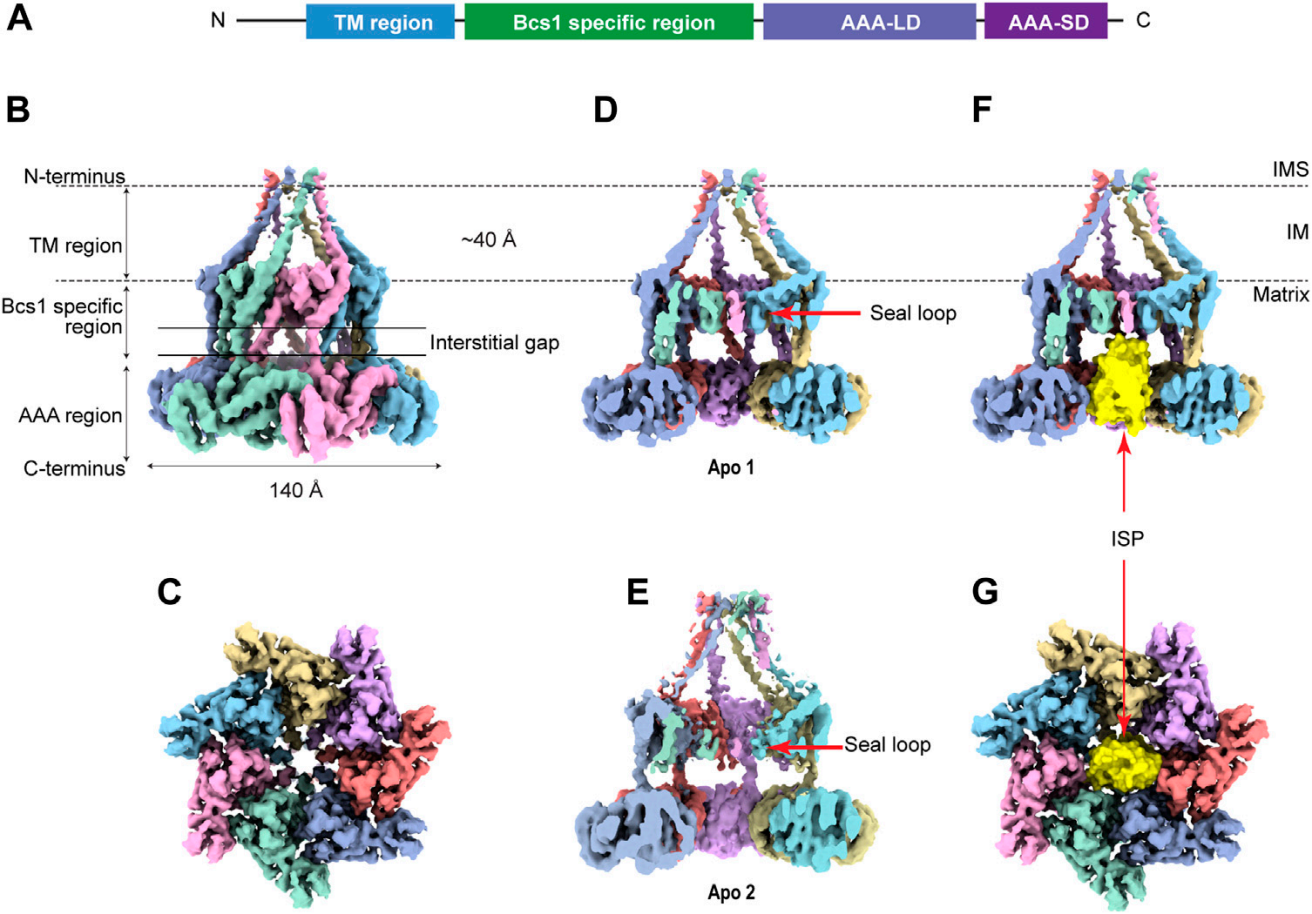
Overview of heptameric Bcs1 in Apo state. **(A)** Schematic diagram showing the domain organization of Bcs1. EM density map of yeast Bcs1 shown in side view **(B)** and top view **(C)** from the matrix side. **(D)** Cut side view of yBcs1 in Apo 1 state with a conical cavity in the mitochondrial inner membrane and a cylindric cavity in the matrix. The two cavities are separated by the middle seal loops (EMD-10193). **(E)** Cut side view of yBcs1 in Apo 2 state where the middle seal loops were pointing towards the matrix thus connecting the two cavities (EMD-10194). The cylindric cavity in the matrix can accommodate a folded ISP-ED (shown in yellow), as is shown in cut side view **(F)** and top view **(G)**.

Bcs1 belongs to the AAA-ATPase protein family that features a conserved AAA-ATPase domain at the C-terminus and a unique N-terminal region for its specific function (Figures 2A, B). The N-terminal region could be further divided into a single transmembrane helix (TM) that anchors Bcs1 on the mitochondrial IM, and a Bcs1-specific domain that resides in the matrix with the ATPase domain. The C-terminal ATPase domain bears sequence similarity to AAA family proteins, with a typical nucleotide binding pocket formed at the interface between the large subdomain (AAA-LD) and small subdomain (AAA-SD). The AAA-ATPase domain harness the energy released from ATP hydrolysis to power the specific function of the protein (Gates and Martin, 2020).

**FIGURE 3:**
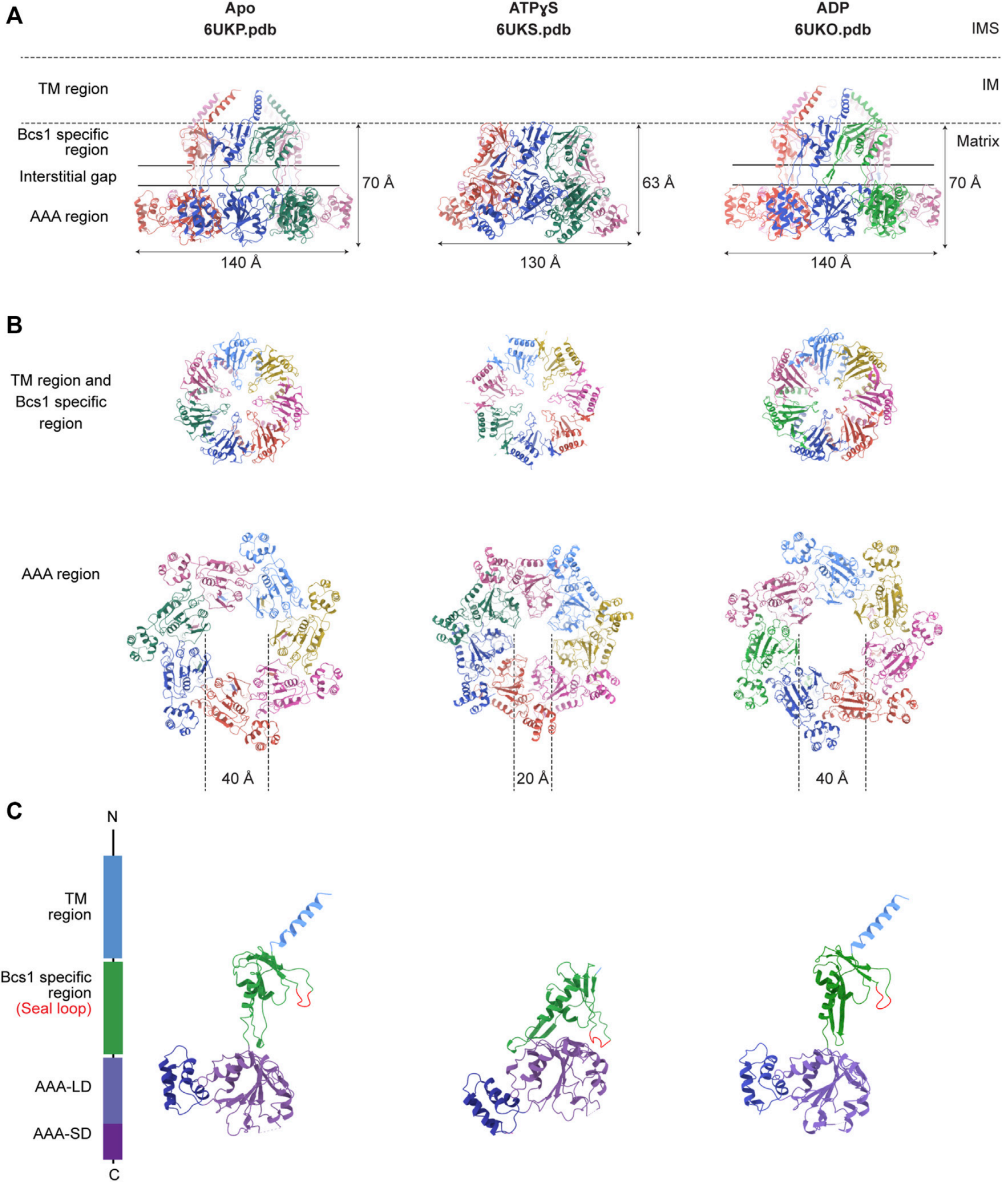
Nucleotide-dependent conformational changes of Bcs1. Ribbon diagram of mBcs1 structures determined in Apo, ATPγS and ADP states respectively, from left to right. **(A)** Side view of the heptameric Bcs1 ring. **(B)** Top view from the matrix side of the heptameric association of Bcs1-specific region and AAA region. **(C)** Isolated Bcs1 protomer models in different nucleotide states highlighting the domain organization. Bcs1 can be divided into TM region, Bcs1-specific region and the AAA region, and the AAA region can be further divided into a large subdomain (AAA-LD) and a small subdomain (AAA-SD). The nucleotide binding pocket lies in the interface between AAA-LD and AAA-SD.

Recently, structures of mouse and yeast Bcs1 (mBcs1, yBcs1) have been determined using Cryo-EM and X-ray crystallography in different nucleotide bound states (Kater et al., 2020; Tang et al., 2020), and both form a homo-heptameric ring with a central pore regardless of the nucleotide states (Figure 2; Figure 3). The rare heptameric assembly of Bcs1 is quite unique compared to the canonical AAA proteins which mostly form homo-hexameric rings. The yeast and mouse Bcs1 overall structures closely resemble each other with differences in certain aspects, thus the features discussed below will apply to both unless otherwise specified.

### 3.1 Structures of Apo Bcs1

In Apo state, the heptameric assembly of Bcs1 has an overall shape of a mushroom when viewed from the matrix side along the central 7-fold axis. The molecule can be divided into three regions along the symmetry axis: the TM region, the Bcs1-specific region and the AAA region; they are similar in height and each measured about 30–40 Å. Within the Bcs1-specific region that connects the AAA region, there is a less-dense part, about 15 Å in height, which is defined as interstitial gap (Figure 2B) (Tang et al., 2020; Xia, 2021). At the mushroom cap, there is a large cylindrical cavity formed by the ATPase domain accessible from the matrix side (Figures 2C–E). This cylindrical cavity has an average diameter of ∼35 Å, a depth of 40 Å, and an estimated size of 28,000 Å^3^ (Tang et al., 2020), which is large enough to accommodate a fully folded ISP-ED (Figures 2F, G). This is consistent with the observation that Bcs1 can interact with ISP-ED in the absence of nucleotides (Wagener et al., 2011; Wagener and Neupert, 2012). On the opposite side of the matrix cavity, there is a smaller conical cavity encircled by the TM helices in the IM. In yBcs1, the TM helices extend through the entire IM and intersect at the intermembrane space side (IMS), thus seal the conical cavity from the IMS side. While in Apo mBcs1 (PDB:6UKP), only half of the TM helices were modelled (Figure 3). The two cavities are separated by the middle seal loops in the Bcs1-specific domain, reaching to the center of the pore.

It is worth noting that there are two Apo structures of yBcs1 obtained from 3D classification and refinement of one Cryo-EM dataset, named Apo1 and Apo2 respectively; their major differences lie in the arrangement of the seal loop in the Bcs1-specific region. Similar to mBcs1, yBcs1 in Apo1 state has the 7 seal loops tightly packed together on the membrane plane that separates the two cavities (Figure 2D), while yBcs1 in Apo2 state has the seal loops pointing away from membrane plane towards the matrix, which consequently opens up the middle seal pore and connects the two cavities (Figure 2E) (Kater et al., 2020).

### 3.2 Structure of Bcs1 with bound ADP

It has been shown that Bcs1 migrates differently in different nucleotide states on a Blue-Native PAGE (Hinson et al., 2007; Wagener et al., 2011; Tang et al., 2020), with the Apo state and the uniform ADP-bound state migrating slower compared to the uniform ATPγS-bound state, suggesting nucleotide-dependent conformational changes that alter its apparent molecular size. The distinct conformations were revealed in detail when mBcs1 structures were determined with uniform ATPγS bound (PDB:6UKS), and ADP bound (PDB:6UKO) (Tang et al., 2020). The yBcs1 structure with uniform ADP bound was also determined (PDB:6SH3) (Kater et al., 2020).

For both mBcs1 and yBcs1, their structures for the ADP state are very similar to those of Bcs1 in the Apo state (Apo1 state for yBcs1), which features a large substrate-binding cavity in the AAA region opening towards the matrix, compatible with ISP-ED binding; and an interstitial gap between the Bcs1-specific region and the AAA region. The substrate-binding cavity is tightly sealed from the IM side by the middle seal loops in the Bcs1-specific domain. The TM helices in mBcs1 are partially built, while in yBcs1 they are complete. The close resemblance between Bcs1 in Apo state and in ADP state also explains their similar migration patterns in the Blue-native PAGE and their ability to bind ISP (Hinson et al., 2007; Wagener et al., 2011; Tang et al., 2020).

### 3.3 ATPγS binding induced conformational changes in Bcs1

The ATPγS-bound state is only available for mBcs1 (Tang et al., 2020) (Figure 3). Upon interaction with ATPγS, mBcs1 underwent conformational changes to a more compact bullet shape compared to the Apo/ADP state. While the TM helices could not be determined and were not modelled, the overall height of the matrix region (Bcs1-specific domain and AAA domain) reduced from 70 Å in the Apo/ADP state to 63 Å in the ATPγS state (Figure 3A). Especially, the interstitial gap between Bcs1-specific region and the AAA region disappeared. The cylindrical cavity formed by the ATPase domain shrank by two thirds in size to a diameter of 20 Å and a depth of 25 Å (Figure 3B), which could no longer accommodate the folded ISP-ED (Tang et al., 2020). Nonetheless, it has been shown that in the presence of AMP-PNP, Bcs1 could bind the N-terminal segment of the ISP presumably after the translocation of the ISP-ED (Wagener et al., 2011). The fact that in the ATPγS-bound state, the TM helices could not be modelled due to poor density suggests a lack of interaction in the TM region, which may open up the seal to the IMS (Figures 3A, C) and allow ISP-ED to pass through. Thus, the ATPγS state is deemed as a post-translocation state (Tang et al., 2020).

## 4 The underlying translocation mechanism

The translocation of ISP-ED is accomplished through ATP hydrolysis-powered conformational changes of Bcs1. The biochemical and structural data of Bcs1 in different nucleotide states provided valuable insights into its translocation mechanism. Unlike the canonical AAA proteins with hexameric association, the heptameric assembly affords Bcs1 a large enough cavity for substrate entry and translocation. Based on biochemical and structural analyses, there are two potential binding sites on Bcs1 for ISP, one is in the AAA domain that is selectively occupied by ISP-ED from the matrix side when Bcs1 is in Apo/ADP state, the other one is present inside the IM accessible from the IMS side that interacts with the N-terminus of ISP after the translocation of ISP-ED, when Bcs1 is in the ATPγS state (Wagener et al., 2011; Kater et al., 2020; Tang et al., 2020).

**FIGURE 4:**
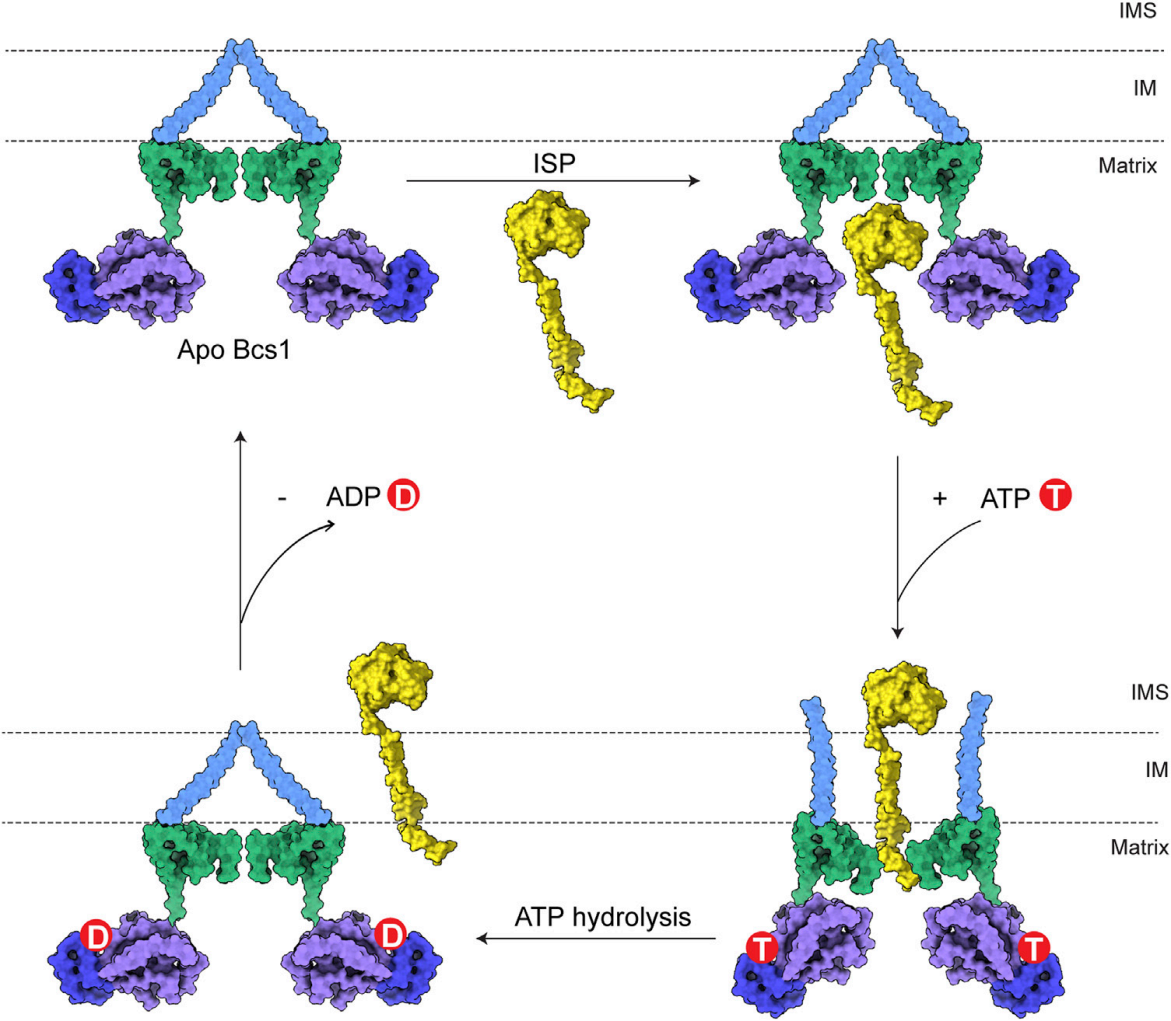
Model for translocation of ISP by Bcs1. The TM region, Bcs1-specific domain, AAA-LD and AAA-SD of Bcs1 are colored in cyan, green, purple and blue, respectively. In a translocation cycle, Bcs1 populates two major conformations: Apo/ADP state and ATP state. (1) In Apo/ADP state, fully folded ISP-ED in mitochondria matrix enters the substrate-binding cavity in Bcs1 AAA domain. (2) Upon interaction with ATP, Bcs1 undergoes conformational change to the more compact ATP state. The substrate-binding cavity contracts and pushes ISP-ED to the IMS, while the N-terminal region of ISP remains in the Bcs1 central pore. (3) Subsequent ATP hydrolysis resets Bcs1 back to the ADP/Apo state, which also abolishes the interaction between Bcs1 central pore and the N-terminus of ISP. The entire ISP is released from Bcs1 for the assembly of bc_1_ complex.

Therefore, a complete translocation cycle of ISP-ED by Bcs1 could be depicted as follows: (1) In Apo/ADP state, fully folded ISP-ED in mitochondria matrix enters the large substrate-binding cavity in Bcs1 AAA domain. (2) Upon interaction with ATP, Bcs1 protomers go through conformational changes to ATPγS state and push ISP-ED to the IMS, while the N-terminal region of ISP remains in the Bcs1 central pore. (3) Subsequent ATP hydrolysis resets the Bcs1 back to the ADP/Apo state, which also abolishes its interaction with the N-terminal region of ISP and releases the entire ISP for the assembly of the cyt bc_1_ complex (Figure 4).

Despite all the progress made on understanding the biogenesis of the mitochondrial cyt bc_1_ complex and the role Bcs1 plays in it, there are still important questions unanswered. First of all, it remains unclear how the seven subunits of Bcs1 coordinate with one another during the translocation process. For the substrate-binding cavity in the AAA region to accommodate the folded ISP-ED, Bcs1 has to be in either uniform ADP state or Apo state. However, in subsequent steps where ATP binding and hydrolysis cause major conformational changes of Bcs1, it is unclear if the transition occurs simultaneously among the seven subunits or sequentially around the ring. Canonical hexameric AAA proteins such as p97 (Cooney et al., 2019; Pan et al., 2021) and ClpAP (Lopez et al., 2020) follow the sequential mechanism where subunits within the ring hydrolyze ATP one by one, causing co-existing nucleotide states and a spiral conformation (Gates and Martin, 2020). Considering Bcs1 is a non-canonical AAA protein with heptameric assembly and a folded protein substrate, it remains to be seen if it is an exception. Secondly, how the entire ISP gets released from the Bcs1 at the end of the translocation cycle remains unknown. Post translocation of ISP-ED, the N-terminal region of ISP remains in the Bcs1 central pore. The N-terminal region of ISP is composed of a TM helix which is about 35 residues long, and a matrix region which could have 30 residues in yeast or over 100 residues in human. It has to either exit through the central pore of Bcs1 to the IMS and re-insert into the IM, or diffuse along the IM after the Bcs1 ring laterally opens up. Thirdly, the proton motive force across the IM generated by mitochondrial respiration must be preserved at all times during the translocation process. The detailed mechanism of ISP translocation and release by Bcs1 without disrupting the membrane permeability remains to be uncovered.

## 5 Bcs1 as a potential drug target for microbial infections

Most targets for antibiotics against bacterial infections take advantage of the evolutionary long distance between bacteria and animals, making it one of the most successful treatment options in modern medicine. Microbial infections caused by fungi or oomycetes, both in agriculture and in animals, are difficult to treat as the pathogens are much closer to their hosts evolutionarily and share more common biological pathways. This awkward situation is reflected by the fact that only a handful classes of antifungals are available for use in medicine, which is in sharp contrast to the numerous antibiotics against bacterial infections. Therefore, a successful drug candidate should meet many criteria including the following two: (1) its inhibition leads to irreversible harm to the pathogen, and (2) ideally, the target should be unique to the pathogen to avoid off-target effect to its hosts.

### 5.1 Bcs1 is connected to the function of the cellular respiratory chain

**FIGURE 5:**
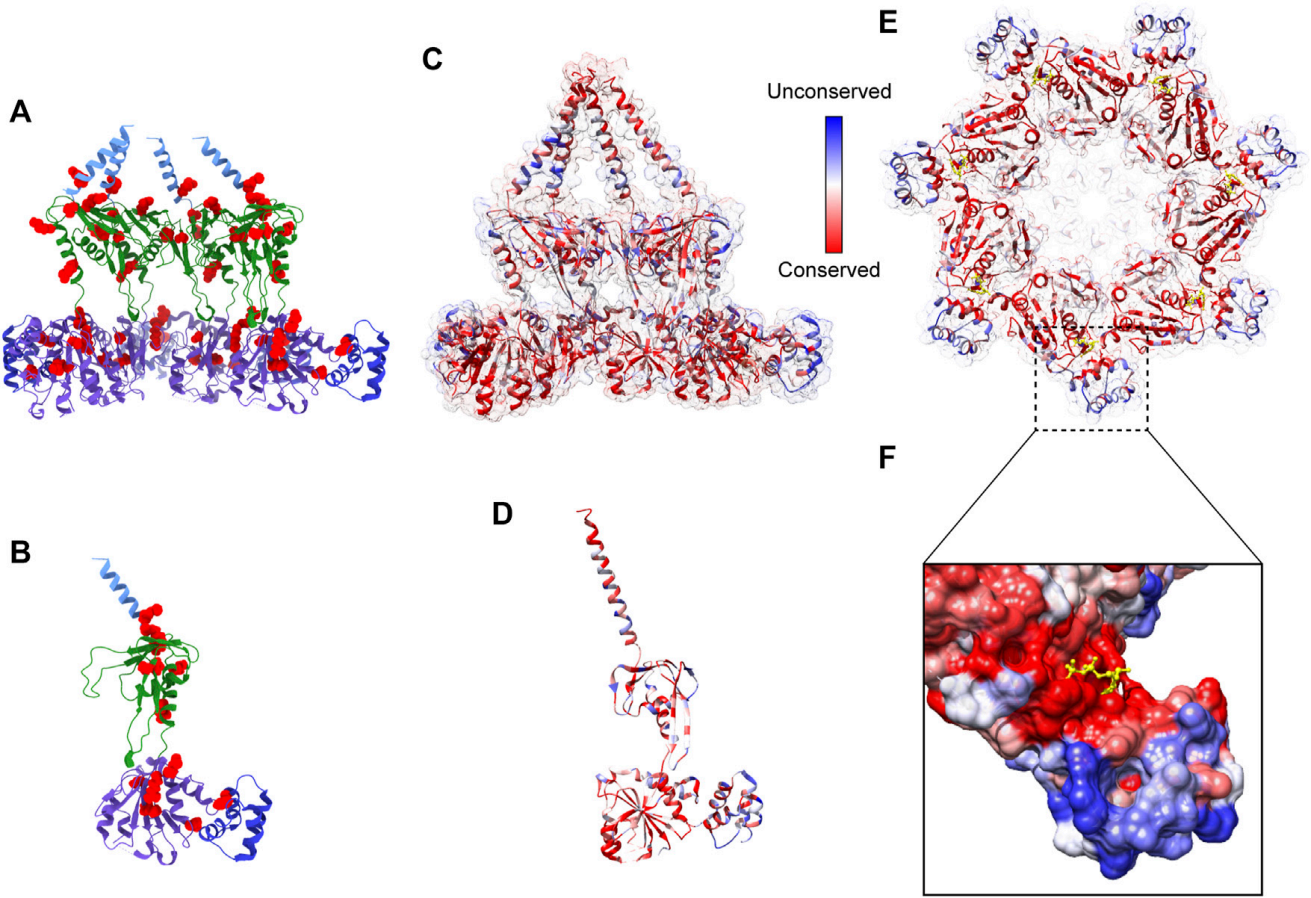
Pathogenic mutations on Bcs1 and potential target for fungicide design. **(A, B)** Pathogenic mutations of BCS1L are mapped onto the ribbon diagram of mBcs1 as red spheres. The TM region, Bcs1-specific domain, AAA-LD and AAA-SD of Bcs1 are colored in cyan, green, purple and blue, respectively. Cut side view is shown in **(A)** and Bcs1 protomer is shown in **(B)**. **(C–E)** Ribbon diagram of mBcs1 colored by sequence conservation, with most conserved residues colored in red and the least conserved residues in blue. The cut side view and bottom view of Bcs1 ring are shown in **(C)** and **(E)** respectively, and Bcs1 protomer is shown in **(D)**. **(F)** Zoom in view of an unconserved pocket in AAA-SD in the close vicinity of nucleotide binding pocket of Bcs1. The bound ADP is shown as yellow sticks. The surface of Bcs1 is also colored by sequence conservation.

Although a complete inactivation of Bcs1 in yeast was accomplished, allowing its role in Complex III assembly to be uncovered, Bcs1 gene knock-out in animals has not been reported. Based on the yeast studies, full inactivation of Bcs1L in animals is likely to be lethal, making it challenging to study in higher organisms. Nevertheless, pathogenic Bcs1L mutations identified in clinical cases (De Meirleir et al., 2003; Hinson et al., 2007; Kasapkara et al., 2014; Fernandez-Vizarra and Zeviani, 2015; Olahova et al., 2019; Guo et al., 2022) point to the vulnerable regions on Bcs1 that are important to maintain its proper function. If these regions are perturbed, there might be severe consequences resembling the clinical symptoms of the mutations, which also makes them potential targets for drug intervention. By mapping the pathogenic mutations on mBcs1 structure, which shares 94% sequence identity with human BCS1L (Table 1), it is revealed that the pathogenic mutations are clustered in Bcs1-specific region and the AAA-ATPase region (Figures 5A, B). More specifically, the severe GRACILE syndrome is frequently caused by mutations in Bcs1-specific region, while the milder Björnstad syndrome is linked to mutations in the AAA region. Combined with the structural information on Bcs1 and available phenotypes of pathogenic mutations, it has been proposed that the structural integrity of Bcs1-specific region is critical for persevering the proton motive force across the IM, and the fetal GRACILE syndrome could be the result of proton leakage caused by defective Bcs1-specific domain on top of the failed cyt *bc*_1_ complex assembly (Tang et al., 2020). Thus, using Bcs1 as a target could potentially interfere with both the activity of Complex III in the respiratory chain and the function of mitochondrial proton motive force across IM.

### 5.2 Identifications of unique features that distinguish Bcs1 of pathogens from hosts

Although it is considered unconventional or even dangerous to target mitochondrial respiratory chain because it is a shared and conserved pathway between pathogens and hosts (Table 1), exceptions to this rule exist. For example, the FDA approved anti-malaria drug atovaquone specifically targets the Complex III of the pathogen’s respiratory chain (Baggish and Hill, 2002). Atovaquone is shown to be safe for prophylactic use in humans. Structural study of famoxadone, a Complex III specific antifungal widely used in crop disease control, revealed organism-specific binding to the active site with a IC_50_ value 400 times smaller against bacterial cyt *bc*_1_ when compared to that against bovine mitochondrial complex (Gao et al., 2002). Thus, it is possible to identify a unique locus in the target, which gives a significant difference between pathogen and host even the target is part of the shared pathway.

**FIGURE 6:**
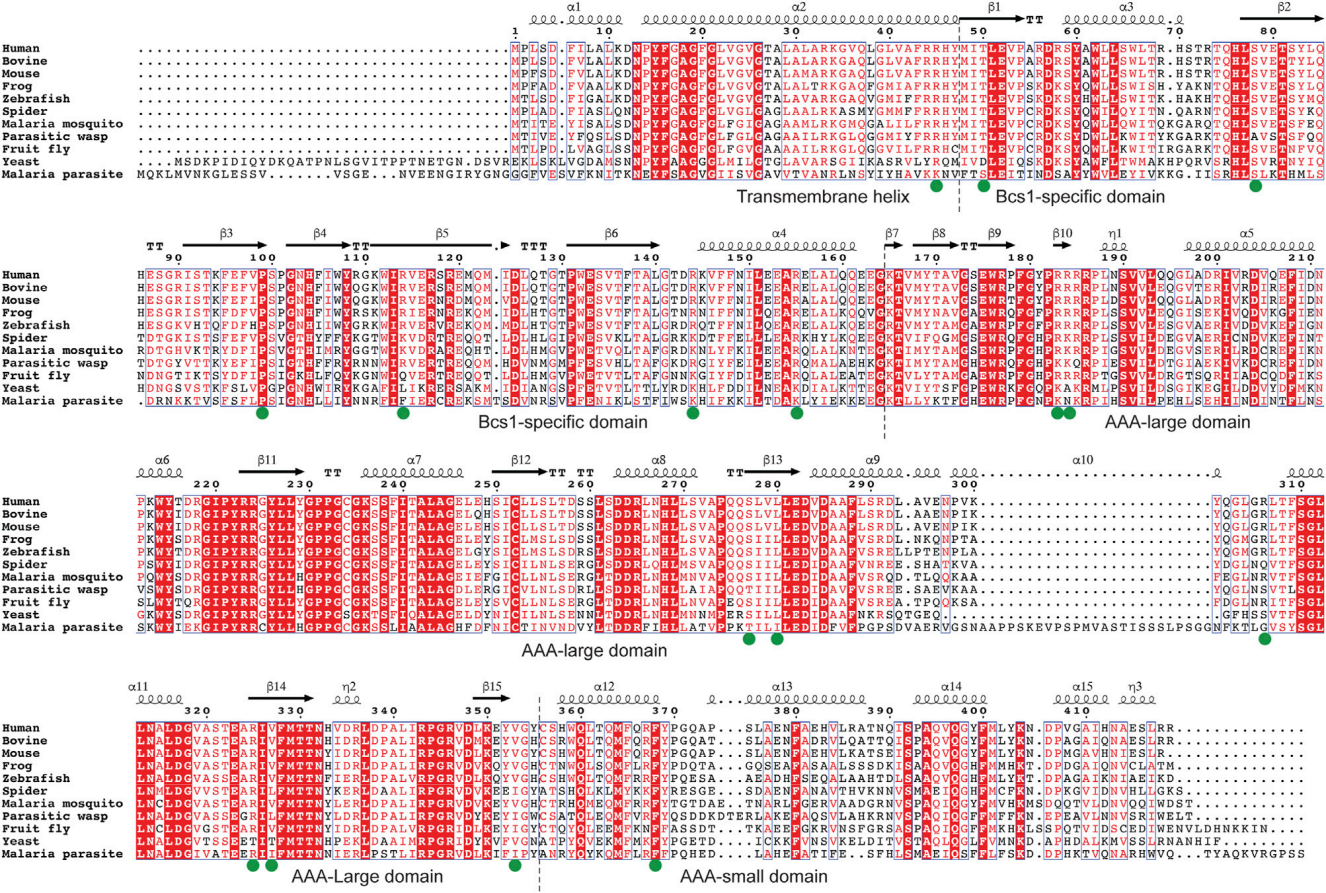
Multiple sequence alignment of Bcs1. Residues with pathogenic mutations implicated in diseases such as GRACILE syndrome, Björnstad syndrome and bc_1_ complex deficiencies are marked with a green dot. Protein sequences were retrieved from the Uniprot with the accession numbers shown in parentheses: Human (Q9Y276), Bovine (Q5E9H5), Mouse (Q9CZP5), African clawed frog (Q7ZTL7), Zebrafish (Q7ZV60), House spider (A0A2L2YIU0), African Malaria Mosquito (A0A182RX69), Parasitic wasp (A0A7M7PZM7), Fruit fly (Q9VL22), Baker’s yeast (P32839), Malaria parasite P. vivax (A0A564ZXW0).

To find such locations on Bcs1-specific and AAA regions suitable for drug targeting, a multisequence alignment of Bcs1 from difference species is provided (Figure 6) and mapped onto yBcs1 structure (Figures 5C–E). In the close vicinity of the nucleotide-binding site, there is a pocket in the AAA-SD that appears to be highly divergent in the sequence alignment (Figure 5F). Potentially, a molecule that specifically targets the ATPase site while having a tail stretching to explore this pocket in pathogenic fungal Bcs1 could block the ATP binding and hydrolysis, thus serve as a fungicide with sufficient specificity to the pathogen. Alternatively, molecules that bind to the interstitial gap in the Bcs1-specific region may be able to block the overall conformational change of Bcs1 upon ATP binding, thus have the Bcs1 stuck in Apo/ADP conformation and unable to finish its translocation cycle.

Since Bcs1 is found in nearly all eukaryotes, it could also be targeted in other eukaryotic pathogens. For instance, both malaria parasite and its transmission vector African malaria mosquito have Bcs1 orthologues which share a sequence identity of 39% and 60% with human BCS1L, respectively (Table 1; Figure 6). Small molecules that are tailored to specifically target the pathogenic Bcs1 could be designed to control these eukaryotic pathogens.

### 5.3 Methods that can be employed to identify Bcs1 inhibitors

Currently, there are two *in vitro* assays of Bcs1 that can be employed for screening inhibitor libraries. One is the ATPase assay that measures ATP hydrolysis by isolated Bcs1. ATPase assay has been widely used to screen inhibitors for other AAA ATPases such as the human AAA protein p97 (Chou et al., 2014), which was quite successful to bring at least one anti-cancer drug to clinical trial (Tang et al., 2019; Xia and Tang, 2020). Another assay that can potentially be used is based on the conformational change induced by binding of ATP or its analogs. The conformational change of Bcs1 can be detected by the Blue-Native PAGE method as illustrated in the previous publications (Hinson et al., 2007; Wagener et al., 2011; Tang et al., 2020), allowing detection of inhibitors that block this conformational transition. These assays can potentially be automated for high throughput screening (HTS).

With the available high-resolution Bcs1 structures of both mBcs1 and yBcs1 in different conformations, we are also in a position to carry out structure-based *in silico* screen of compound database to select promising candidates. The HTS approach realizes sensitive detection and automatic operation, but its positive rate is low, thus the cost of HTS is relatively high. However, the virtual screening has a relatively high hit rate and inexpensive. By combining the results of virtual screening with experimental screening, the latter can be more targeted, has a higher hit rate, thus shortening the research cycle and reducing the cost of drug development.

## Abbreviations

AAA, ATPases Associated with diverse cellular Activities; Cyt1, yeast cyt c1; cyt c1, cytochrome c1; cyt b, cytochrome b; Fe_2_S_2_, iron-sulfur cluster; HTS, high throughput screening; ISP, Rieske iron-sulfur protein; ISP-ED, extrinsic domain of the Rieske iron-sulfur protein; IM, mitochondrial inner membrane; IMS, mitochondrial intermembrane space; OM, mitochondrial outer membrane; PMF, proton motive force; TAT, twin arginine translocation; TIM, translocase of the inner mitochondrial membrane; TM, transmembrane; TOM, translocase of the outer mitochondrial membrane; mBcs1, mouse Bcs1; yBcs1, yeast Bcs1.
